# Dynamic alterations in the irradiated draining lymph node

**DOI:** 10.1038/s41698-026-01610-y

**Published:** 2026-07-11

**Authors:** Sabrina Reichl, Lisa Kurz, Alba Sanchez Fernandez, Elina Bugar, Irene Vetrugno, Burkhard Ludewig, Martin Pruschy, Irma Telarovic

**Affiliations:** 1 Laboratory for Applied Radiobiology, University of Zurich, Zurich, Switzerland; 2 Department of Radiation Oncology, University Hospital Zurich, Zurich, Switzerland; 3 Institute of Immunobiology, Medical Research Center, Cantonal Hospital St.Gallen, St. Gallen, Switzerland

**Keywords:** Cancer, Immunology, Oncology

## Abstract

Tumor-draining lymph nodes (DLNs) are crucial for the development of anti-tumor immunity upon irradiation. However, in radiotherapy, DLNs are frequently co-irradiated, which impairs their function. Here, we examine the consequences of DLN irradiation on anti-tumor immunity, focusing on the recently described radiotherapy-induced disruption of the CCR7-CCL19/CCL21 axis, which governs tumor-to-DLN trafficking. We use in vivo irradiated murine LNs and in vitro assays to assess irradiation-induced structural and functional changes in the DLNs. DLN-infiltrating lymphocytes and CCL19/CCL21 chemokines were depleted early after irradiation in a dose-dependent manner. Lymphocyte numbers recovered only in DLNs irradiated with 5 Gy or less. Lymphopenia persisted in DLNs irradiated with 15 Gy, with few surviving lymphocytes showing a compensatory increase in proliferation. Irradiation-deregulated secretion of CCL19 could be related to lymphodepletion and/or direct irradiation-induced processes in stromal cells. In conclusion, radiotherapy depletes DLNs, and impairs stromal function and chemokine signaling, thereby compromising anti-tumor immunity.

## Introduction

Radiotherapy, traditionally viewed as an exclusively local treatment modality, has recently been shown to also have systemic immunostimulatory effects. This property has led to the attempts of combining radiotherapy with immune checkpoint inhibitors (ICI) in order to synergistically increase the efficacy of each individual treatment modality^1–3^. Encouraging findings from preclinical research, supported by strong mechanistic evidence^4,5^, led to numerous large clinical trials exploring this approach. However, translating the preclinical successes to clinical practice has proven difficult. Particularly in cases of metastatic disease, most clinical trials have failed to demonstrate a benefit of combining radiotherapy with ICIs^6,7^.

One potentially highly relevant factor contributing to these challenges is the widespread practice of co-irradiating tumor-draining lymph nodes (DLNs) as part of the curative treatment approach^6,7^. DLNs play a pivotal role in the cancer-immunity cycle^8,9^. Tumor antigens taken up by activated dendritic cells (DCs) are transported to DLNs via the lymphatic system, where the unique microenvironment supports the priming of naïve tumor antigen-specific T cells. This process is critical for initiating anti-tumor immune responses and achieving the in-situ vaccination effect of radiotherapy. Therapeutic irradiation of DLNs during radiotherapy, which is necessary to manage early metastatic spread may thereby render these nodes immunologically dysfunctional during a crucial time of mounting anti-tumor immunity. Growing preclinical evidence has increasingly provided evidence to support this hypothesis, in particular in the context of combined radioimmunotherapy^10–12^.

Recently, Telarovic et al. explored the strategy of temporally separating the irradiation of the tumor from the irradiation of lymph nodes (LNs) to preserve the stimulatory effect of tumor irradiation on anti-tumor immunity, while still ensuring the eradication of LN metastases. Using several murine preclinical tumor models, we demonstrated that delaying DLN irradiation preserves the contribution of radiotherapy to the anti-tumor immunity and consequently significantly enhances the efficacy of combining radiotherapy with ICIs, most probably by allowing DLNs to remain functional during the critical phase of immune activation^13^.

CCL19 and CCL21 are homeostatic chemokines produced primarily by LN stromal cells, including fibroblastic reticular cells (FRCs) and endothelial cells in the lymphatic vasculature. These chemokines are essential for maintaining the structure and function of the LN microenvironment, particularly in immune cell trafficking and activation. By binding to the CCR7 receptor expressed on naïve T cells, central memory T cells, and dendritic cells, CCL19 and CCL21 guide these cells into LNs through high endothelial venules (HEVs). They also direct activated dendritic cells carrying antigens toward DLNs via lymphatic vessels, enabling effective antigen presentation and T cell activation. Furthermore, they organize immune cells into functional compartments within the LN, with CCL19 predominantly found in T cell zones and CCL21 along lymphatic conduits^14–16^. Telarovic et al. recently described a disruption of the CCR7-CCL19/CCL21 axis upon irradiation, which correlated with significant changes in the immune cell composition of the irradiated LNs, in particular with a sustained, possibly irreversible lymphopenia^13^. Based on the simultaneously observed structural and quantitative alterations in the stromal cell network of irradiated LNs, Telarovic et al. proposed that the negative effect of LN irradiation on the efficacy of combined radioimmunotherapy might largely depend on irradiation-induced functional impairments in the stromal cell network. The differential radiosensitivity of lymphocytes compared to stromal cells in lymphoid organs has been recognized since foundational studies in the 1970s, providing the basis for understanding how radiation disrupts lymph node function^17,18^.

Given the critical importance of preserving LN function to achieve optimal outcomes in radioimmunotherapy, in this study, we aimed to conduct an in-depth investigation of the previously elusive radiation-induced changes in the LN in order to improve our understanding of the communication between the tumor and the DLN upon irradiation. Telarovic et al. previously highlighted the critical role of the CCR7-CCL19/CCL21 homing axis and the chemokine-producing stromal cells of the LN in response to irradiation. However, detailed insights into the kinetics of the CCL19/CCL21 secretion and its interaction with the observed immune dysfunction following irradiation are lacking. To address this, we irradiated LNs of healthy, non-tumor-bearing mice and quantified the changes within the lymphocyte population at different time points and in response to different doses of irradiation. In addition, we performed multiplex cytokine profiling, as well as an immunohistological examination of the LN architecture.

## Results

### Irradiation induces lymphopenia in the lymph node and reduces CCL19/CCL21 protein levels in a dose-dependent manner

To characterize the T lymphocyte response in LNs to irradiation, axillary, brachial, and inguinal lymph nodes were stereotactically irradiated with increasing doses of ionizing radiation (0–20 Gy) and harvested at an early (2 days) and a delayed time point (9 days) following irradiation. Lymph node composition was analyzed by flow cytometry-based immunophenotyping.

**Fig. 1:**
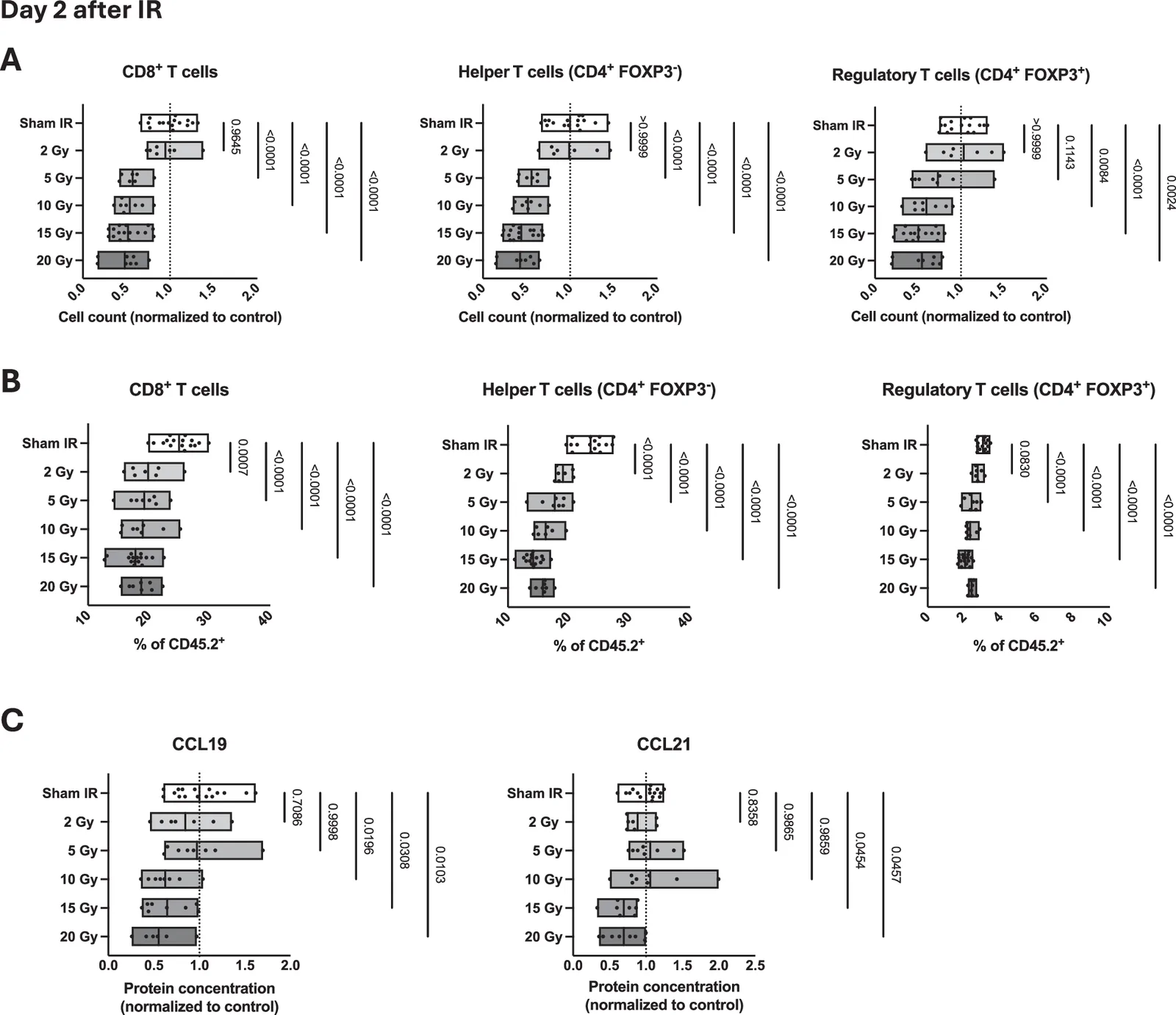
Irradiation induces lymphopenia in the lymph node and reduces CCL19/CCL21 protein levels in a dose-dependent manner. Brachial, axillary, and inguinal LNs on both sides of C57BL/6 mice were irradiated with 2, 5, 10, 15, or 20 Gy and harvested after 2 days. Immune cell composition of the LNs. CD8 + T cells, helper T cells (CD4^+^ FOXP3^-^) and regulatory T cells (CD4^+^ FOXP3^+^), represented by cell counts (**A**) and as a percentage of CD45^+^ cells (**B**). Gating strategy is shown in Supplementary Fig. 1. Absolute cell count values corresponding to panel A are provided in Supplementary Table 3. **C** CCL19 and CCL21 protein concentration in the irradiated LNs, expressed relative to the average value of sham-irradiated mice (white). *n* ≥ 7 mice per group. Each dot represents one individual mouse. Floating bars span from the minimum to the maximum value within each group. Line indicates the mean value. Statistics: one-way ANOVA with Holm–Sidak’s multiple comparisons test.

At the early timepoint, irradiation resulted in a significant reduction in the lymphocyte populations, with a dose-dependent decrease observed across all T cell subgroups (Fig. 1A, B and Supplementary Table 3). Notably, T cells were not only depleted in absolute numbers but also showed a reduction in their relative frequency among CD45.2⁺ cells (Fig. 1B), underscoring their heightened radiosensitivity compared to other immune cells residing within the LN. Among T cell subsets, no differential radiosensitivity was observed.

CCL19 and CCL21 are homeostatic chemokines produced primarily by LN stromal cells and are essential for immune cell trafficking within the cancer-immunity cycle^15,16^. Telarovic et al. previously identified the disruption of the CCR7-CCL19/CCL21 homing axis in the draining lymph nodes of tumor-bearing mice in response to a single high dose of ionizing radiation^13^. Multiplex-based cytokine assessment of the chemokines CCL19 and CCL21 now revealed a dose-dependent decrease of these cytokines in the irradiated lymph nodes following irradiation with increasing doses of ionizing radiation (Fig. 1C). Interestingly, a single dose of 5 Gy already induced significant lymphodepletion in the irradiated lymph nodes. In contrast, the reduction in the levels of CCL19 and CCL21 was detectable only upon irradiation with 10 Gy or above (Fig. 1A-C). These differential, potentially threshold-dose-dependent responses suggest that lower doses of irradiation may primarily impact lymphocyte populations without severely disrupting chemokine signaling.

### Lymph nodes fail to fully recover from irradiation-induced lymphodepletion

**Fig. 2:**
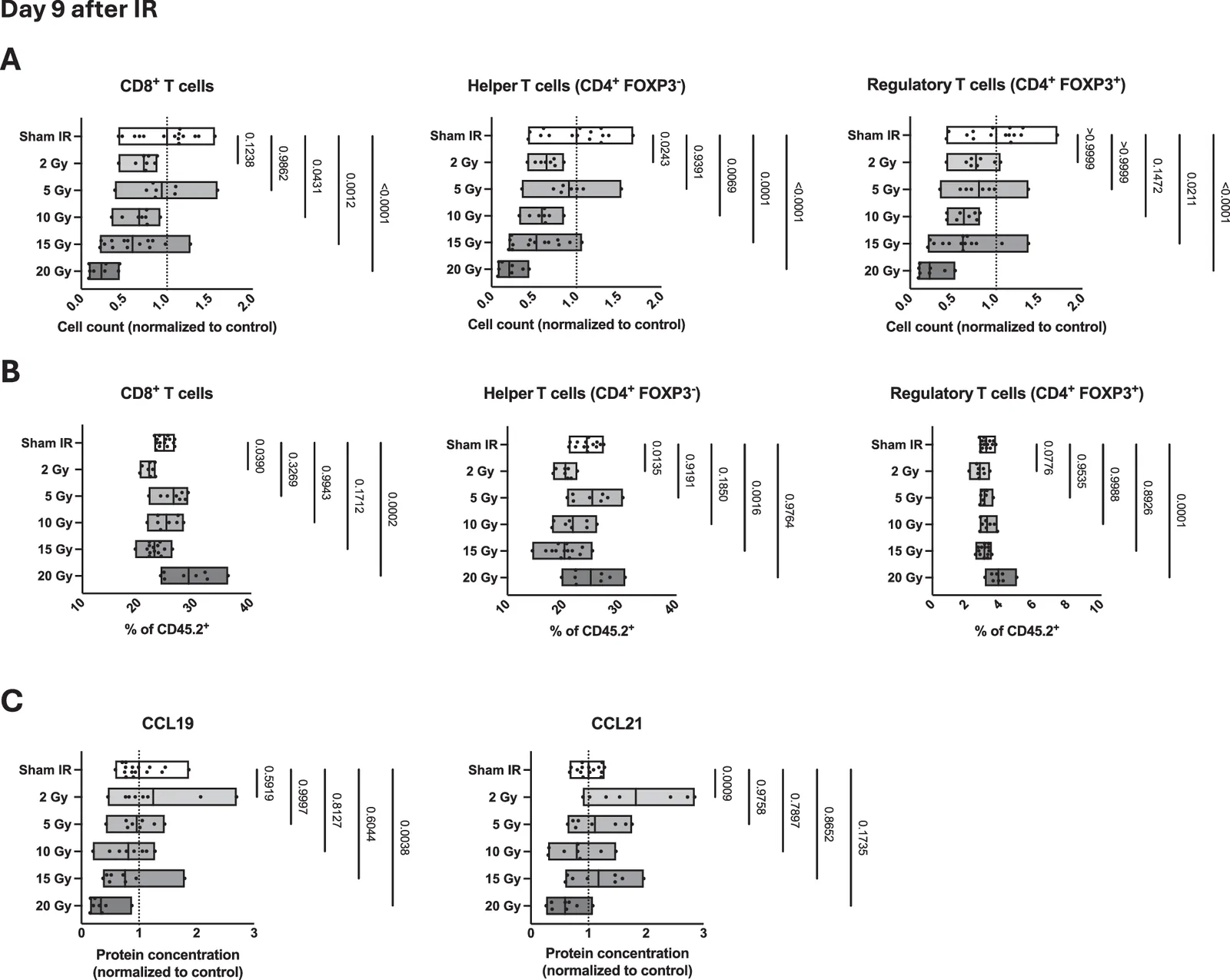
Lymph nodes fail to fully recover from radiation-induced lymphodepletion. Brachial, axillary, and inguinal LNs on both sides of C57BL/6 mice were irradiated with 2, 5, 10, 15, or 20 Gy and harvested after 9 days. Immune cell composition of the LNs. CD8 + T cells, helper T cells (CD4^+^ FOXP3^-^) and regulatory T cells (CD4^+^ FOXP3^+^), represented by cell counts (**A**) and as a percentage of CD45^+^ cells (**B**). Gating strategy is shown in Supplementary Fig. 1. Absolute cell count values corresponding to panel A are provided in Supplementary Table 4. **C** CCL19 and CCL21 protein concentration in the irradiated LNs, expressed relative to the average value of sham-irradiated mice (white). *n* ≥ 7 mice per group. Each dot represents one individual mouse. Floating bars span from the minimum to the maximum value within each group. The line indicates the mean value. Statistics: one-way ANOVA with Holm–Sidak’s multiple comparisons test.

By day 9 post-irradiation, LNs exhibited signs of partial recovery, with the absolute numbers of immune cells rebounding across all major T cell subsets (Fig. 2A, B and Supplementary Table 4). Notably, the disproportional depletion of the T cell subset on day 2 post irradiation (as compared to other CD45.2⁺ immune cells, Fig. 1B) was fully reversed by day 9. At this later time point, the composition of the immune cell infiltrate in the irradiated LNs corresponded to that of the sham-irradiated LNs (Fig. 2B). A partial recovery was also evident in the abundance of chemokines CCL19 and CCL21 (Fig. 2C). However, restoration of both immune cell composition and chemokine levels remained incomplete in a dose-dependent manner. On day 9 after irradiation, the most pronounced, persistent lymphopenia with accompanying CCL19/21 reduction was still present in the lymph nodes exposed to 20 Gy (Fig. 2A–C).

### Irradiation does not diminish key cytokines involved in lymphocyte homeostasis within the lymph node

**Fig. 3:**
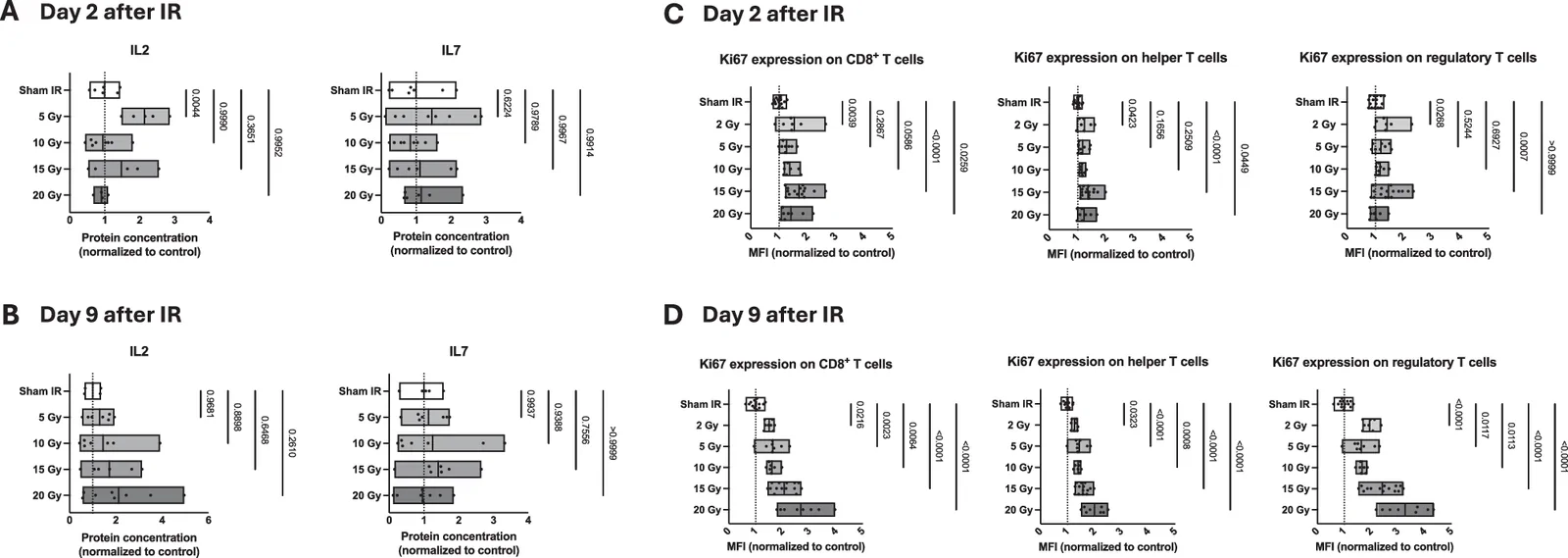
Irradiation does not diminish key cytokines involved in lymphocyte homeostasis within the lymph node. Brachial, axillary, and inguinal LNs on both sides of C57BL/6 mice were irradiated with 2, 5, 10, 15, or 20 Gy and harvested after 2 days (**A**, **C**) or 9 days (**B**, **D**), respectively. **A**, **B**) IL2 and IL7 protein concentration in the irradiated LNs, expressed relative to the average value of sham-irradiated mice (white). *n* ≥ 4 mice per group. Each dot represents one individual mouse. **C**, **D**) Ki67 expression on T cell subgroups, expressed as the geometric mean of the fluorescence intensity (MFI), normalized to the average MFI value of sham-irradiated mice (white). *n* ≥ 6 mice per group. Each dot represents one individual mouse. Floating bars span from the minimum to the maximum value within each group. The line indicates the mean value. Statistics: one-way ANOVA with Holm–Sidak’s multiple comparisons test.

The immune system maintains a tightly regulated homeostasis within the lymphocyte compartment. In response to lymphocyte depletion, it rapidly activates compensatory mechanisms aimed at restoring lymphocyte numbers to basal levels by promoting proliferation and survival. Key mediators involved in this homeostatic response in the lymph node include the survival- and proliferation-promoting cytokines IL2 and IL7^19–21^. In contrast to our expectation, the levels of IL2 and IL7 did not differ between the irradiated and the sham-irradiated LNs (Fig. 3A, B). In fact, in the LNs irradiated with 5 Gy, the level of IL2 even increased above the basal level, thus suggesting at least a partial preservation of the compensatory response (Fig. 3A). In support of these findings, we also observed a dose-dependent increase in the expression of the proliferation marker Ki-67 within all T cell subgroups as early as day 2 post-irradiation (Fig. 3C). On day 9 after irradiation, Ki-67 expression further increased (Fig. 3D). These results suggest that the compensatory proliferative response aimed at restoring homeostasis in the lymphodepleted LNs remains at least partially preserved upon irradiation.

### Irradiation impairs LTα1β2-induced CCL19 protein secretion in lymph node stromal cells

**Fig. 4:**
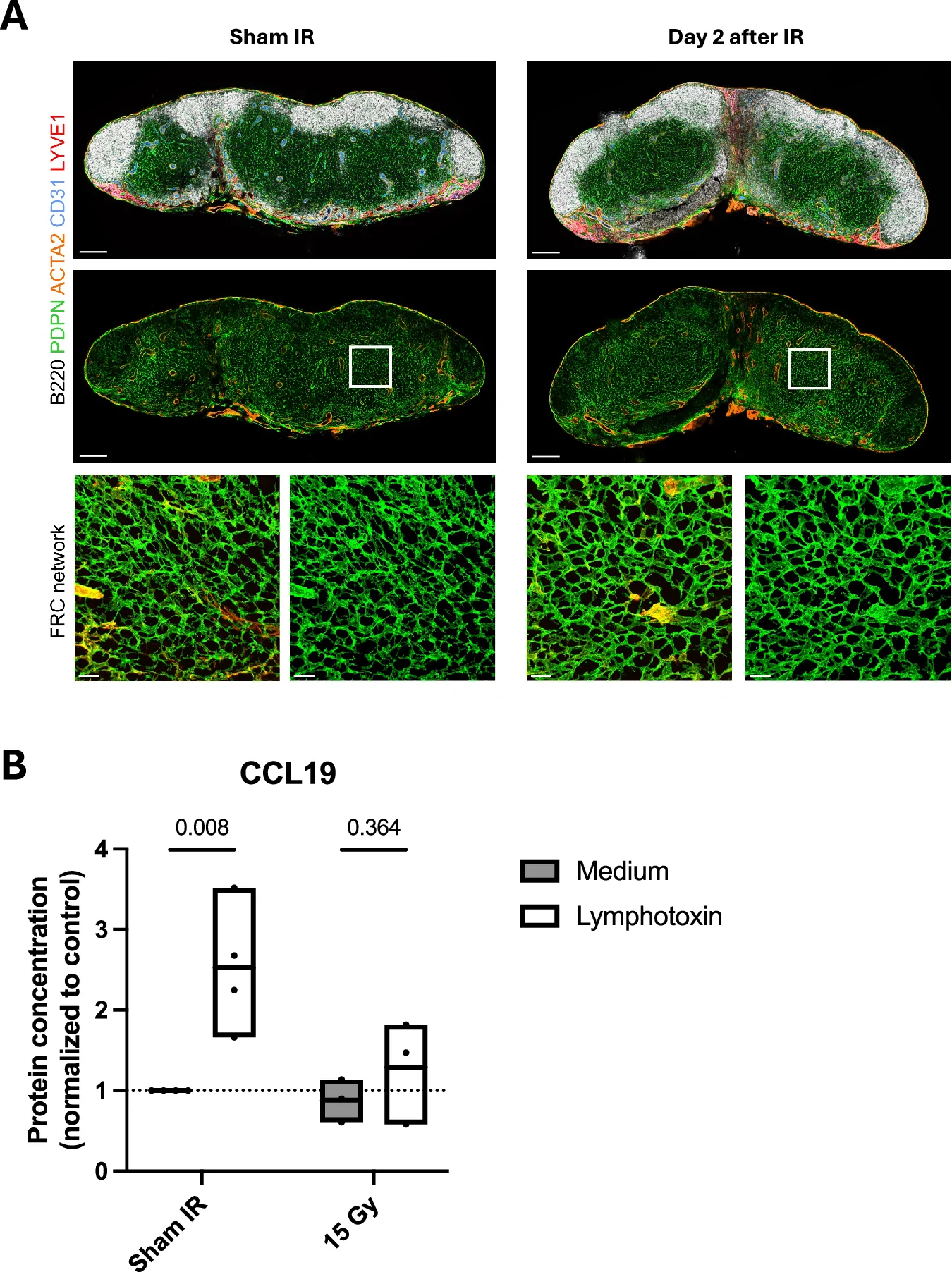
Irradiation impairs LTα1β2-induced CCL19 protein secretion in lymph node stromal cells. **A** Brachial, axillary, and inguinal LNs on both sides of C57BL/6 mice were irradiated with 15 Gy and harvested after 2 days. Fluorescence microscopy was performed on sections from sham-irradiated (left column) and irradiated LNs (right column). Sections were stained for B220 (white), podoplanin (PDPN) (green), actin alpha 2 (ACTA2) (orange), CD31 (blue) and LYVE1 (red). Bottom row shows magnified views of the areas outlined by white squares. Images are representative of *n* = 6 mice per group. Scale bars: 200 µm (top and middle row) and 20 µm (bottom row). **B** Isolated LN stromal cells were treated with lymphotoxin-α1β2 (white) or serum-reduced medium alone (gray) and irradiated with 15 Gy. Shown is the CCL19 protein concentration in the supernatant 48 h post-irradiation, expressed relative to the average value of sham-irradiated, serum-treated cells. Each dot represents one individual experiment; *n* = 3–4. Floating bars span from the minimum to the maximum value within each group. The line indicates the mean value. Statistics: two-sided unpaired *t*-test.

Telarovic et al. previously reported that LN irradiation induces structural and phenotypic alterations in stromal cells by day 9 post-irradiation, including reduced frequencies of FRCs and lymphatic endothelial cells (LECs), disruption of the FRC network, and upregulation of activation markers such as ICAM-1 and VCAM-1^13^. To determine early IR-induced responses, LN stromal populations were analyzed at day 2 post-irradiation. At this early time point, the cell counts and frequencies of FRCs and other stromal subsets remained comparable to those in sham-irradiated LNs (Supplementary Fig. 2A, B). However, expression of activation markers such as ICAM-1, VCAM-1, and SCA-1 was already significantly increased, particularly on FRCs (Supplementary Fig. 2C), indicating that stromal cell activation is an early response to irradiation, which precedes quantifiable changes in population size. Immunofluorescence analysis of entire LNs on day 2 post-irradiation did not show major structural changes (Fig. 4A).

To investigate the interplay between the stromal cells and CCL19 secretion in response to irradiation, primary (FRC) stromal cells were isolated from murine LNs, cultured in vitro and stimulated with lymphotoxin-α1β2 (LTα1β2), the canonical ligand of the lymphotoxin-β receptor (LTβR). LTα1β2 is a key signal provided by activated T cells in vivo and drives the differentiation of stromal precursors into CCL19-producing FRCs^15^. We used exogenous LTα1β2 stimulation to mimic the natural environment of the lymph node in vitro and determined the secretion of CCL19 from lymphotoxin-stimulated FRCs. Secreted CCL19 protein levels were analyzed in culture supernatants 48 hours post-irradiation. As expected, sham-irradiated stromal cells responded to LTα1β2 stimulation with a strong increase in CCL19 secretion. On the contrary, stromal cells exposed to 15 Gy failed to respond to LTα1β2 stimulation (Fig. 4B). A similar response pattern to lymphotoxin stimulation of irradiated FRCs, albeit less pronounced, was observed following irradiation with 5 Gy (Supplementary Fig. 3A). These results suggest that both IR-induced T cell depletion and specific IR-induced processes on the level of FRCs contribute to diminished CCL19 secretion. Analysis of *Ccl19* mRNA expression 24 h post-stimulation with LTα1β2 revealed a trend toward upregulated *Ccl19* transcription in sham-irradiated and no upregulation in irradiated cells (Supplementary Fig. 3B).

### Irradiation induces persistent structural changes in the lymph node

**Fig. 5:**
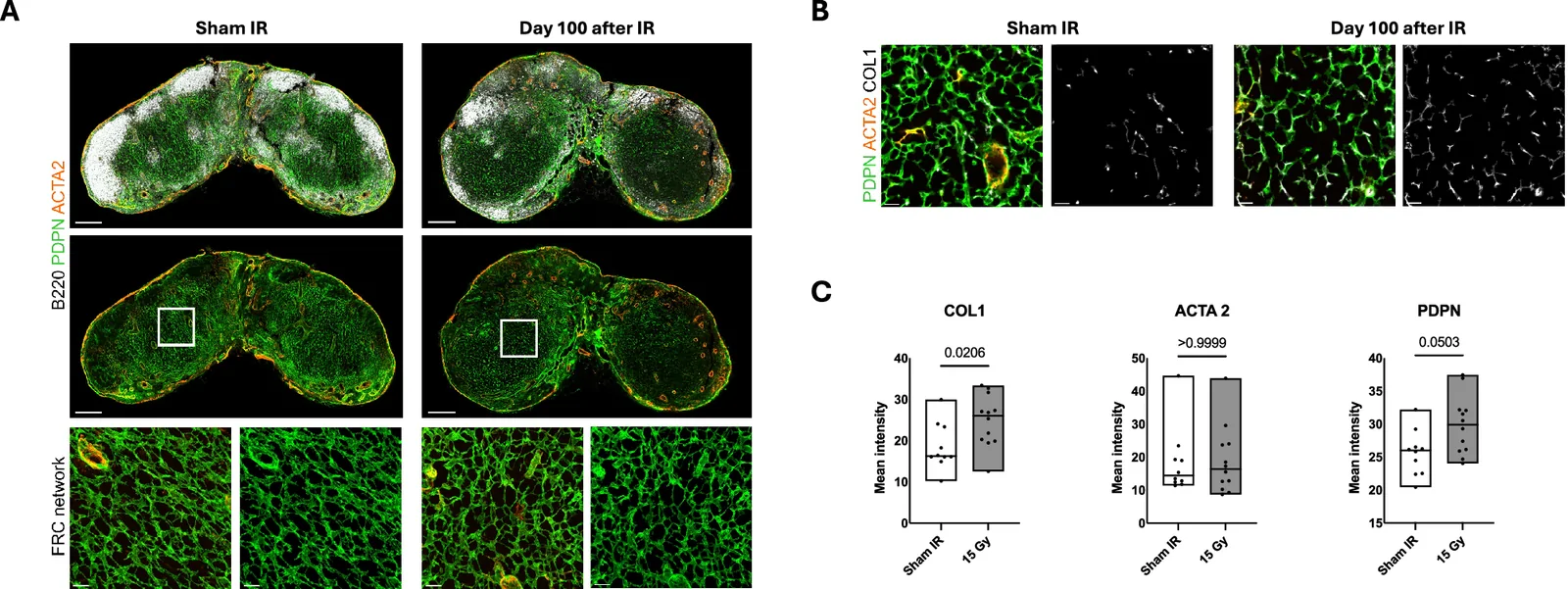
Irradiation induces persistent structural changes in the lymph node. Brachial, axillary, and inguinal LNs on both sides of C57BL/6 mice were irradiated with 15 Gy and harvested after 100 days. **A** Fluorescence microscopy of a section of a sham-irradiated (left column) and an irradiated LN (right column). Sections were stained for B220 (white), podoplanin (PDPN) (green), and actin alpha 2 (ACTA2) (orange). The bottom row shows magnified views of the areas outlined by white squares. Images are representative of *n* = 6 mice per group. Scale bars: 200 µm (top and middle row) and 20 µm (bottom row). **B** Fluorescence microscopy of a section of a sham-irradiated (left column) and an irradiated LN (right column), enlarged in the LN T cell zones. Sections were stained for PDPN (green), ACTA2 (orange), and Collagen 1 (COL1) (white). Images are representative of *n* = 6 mice per group. Scale bar: 20 µm. **C** Quantification of COL1, ACTA2, and PDPN, expressed as the mean intensities. Each dot represents a single LN, derived from *n* = 6 mice per group. Floating bars span from the minimum to the maximum value within each group. The line indicates the mean value. Statistics: two-sided Mann–Whitney test.

To characterize long-term responses in lymph nodes that did not recover from radiation-induced lymphodepletion, LNs were stereotactically irradiated with 15 Gy and examined 100 days following IR. Immunofluorescence staining of LN sections revealed disrupted B cell follicles (Fig. 5A, *top row*), and a reduced density and connectivity of the FRC network, as visualized by podoplanin (PDPN) staining (Fig. 5A, *middle and bottom row*), suggesting enduring structural changes in the LN architecture.

Quantification of structural markers showed a significant accumulation of collagen 1 (COL1), unchanged ACTA2 (alpha-smooth muscle actin) levels, and a modest increase in PDPN expression (Fig. 5B, C), indicating chronic tissue remodeling following irradiation.

**Fig. 6:**
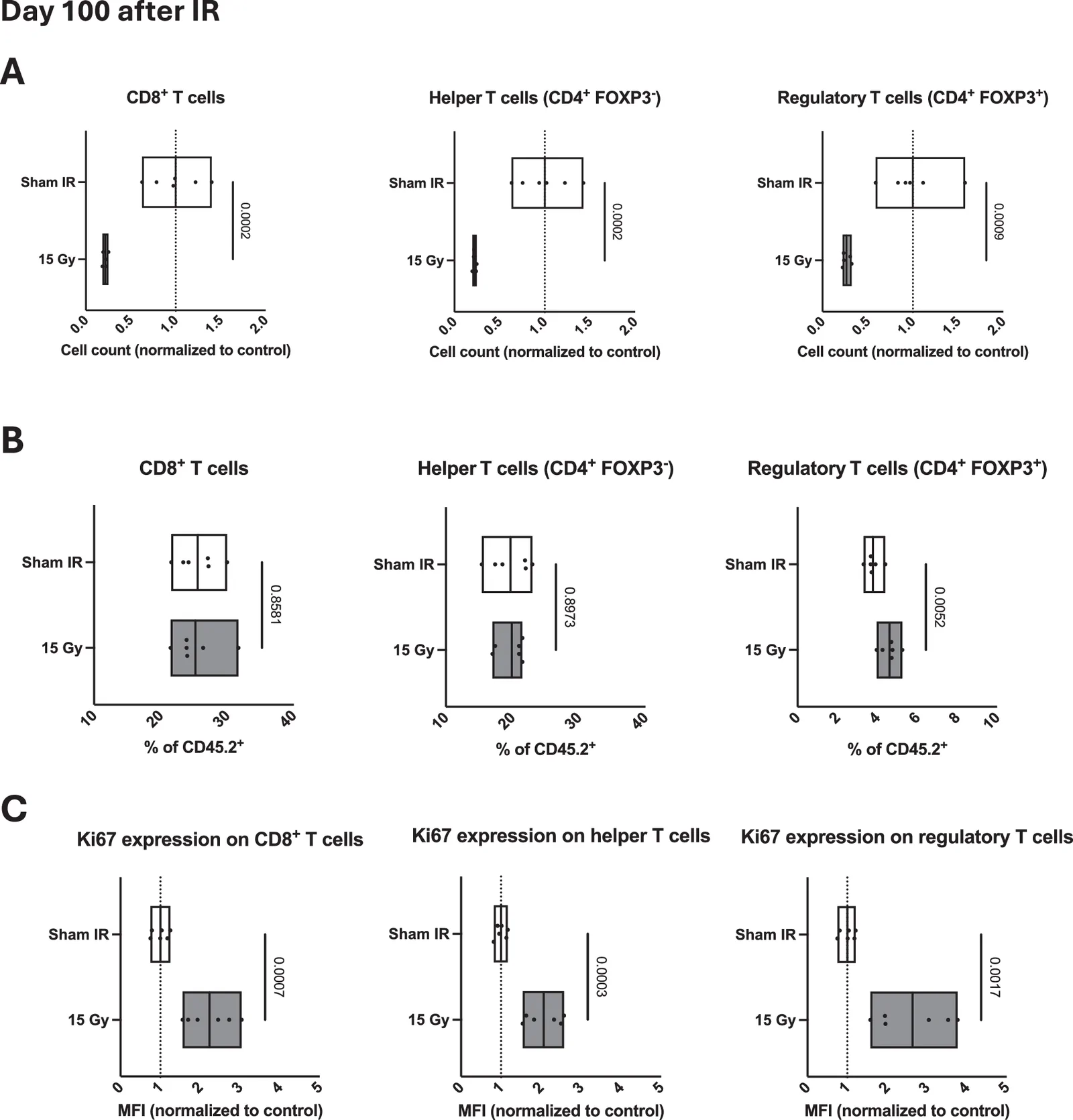
Irradiation with 15 Gy induces long-lasting lymphopenia in the lymph node. Brachial, axillary, and inguinal LNs on both sides of C57BL/6 mice were irradiated with 15 Gy and harvested after 100 days. Immune cell composition of the LNs. CD8 + T cells, helper T cells (CD4^+^ FOXP3^-^) and regulatory T cells (CD4^+^ FOXP3^+^), represented by cell counts (**A**) and as a percentage of CD45^+^ cells (**B**). Gating strategy is shown in Supplementary Fig. 1. Absolute cell count values corresponding to panel A are provided in Supplementary Table 5. **C** Ki67 expression on T cell subgroups, expressed as the geometric mean of the fluorescence intensity (MFI), normalized to the average MFI value of sham-irradiated mice (white). *n* = 6 mice per group. Each dot represents one individual mouse. Floating bars span from the minimum to the maximum value within each group. Line indicates the mean value. Statistics: two-sided unpaired *t*-test.

Interestingly, LNs remained significantly depleted of immune cells at this late timepoint, with a sustained reduction in the absolute numbers of all major T cell subsets on day 100 after irradiation (Fig. 6A and Supplementary Table 5). When analyzed as a proportion of total CD45.2⁺ cells, the frequencies of CD8⁺ and helper T cells were comparable to those in sham-irradiated controls, while regulatory T cells were relatively increased (Fig. 6B), suggesting a differential recovery or survival advantage compared to other immune cell subsets. Unfortunately, and due to technical reasons, no chemo- and cytokines could be analyzed in these lymph nodes. Of note, and despite the strongly reduced cell numbers present on day 100 in the irradiated LNs (Fig. 6A), Ki67 expression remained upregulated across all T cell subgroups (Fig. 6C), reflecting ongoing proliferative activity within the remaining T cell populations in the irradiated LN.

## Discussion

Tumor-draining lymph nodes (DLNs) are critical for initiating anti-tumor immunity, yet their role in determining the outcome of radioimmunotherapy has only recently gained attention on the preclinical and clinical level^3,6,7,13,22^. While radiotherapy can promote immune activation, it may also compromise DLN function, thereby raising concerns about unintended immunosuppression when DLNs are included in the treatment field.

This study extends previous observations by providing quantitative dose thresholds and temporal kinetics of how focal irradiation alters the immune landscape and structural integrity of DLNs. Using a combination of in vivo and in vitro approaches, we demonstrate that irradiation induces a dose-dependent depletion of T cells, followed by impaired chemokine signaling, functional paralysis of stromal cells, and ultimately long-term architectural remodeling. While some regenerative responses emerge early, these are insufficient to restore DLN functionality at moderate-to-high doses. Our findings highlight an irradiation-dose-dependent multi-step breakdown of DLN-mediated immune coordination, which may explain the limited efficacy of radioimmunotherapy when DLNs are co-irradiated.

We show a precise, dose-dependent delineation of lymphocyte depletion across multiple T cell subsets following LN irradiation. Remarkably, lymphopenia in the LN was already observed in response to irradiation as low as 5 Gy, preceding measurable suppression of CCL19 and CCL21 chemokine levels, which only occurred at higher doses of ionizing radiation (≥10 Gy). This tiered response suggests that lower radiation doses primarily affect lymphocyte populations, while largely sparing the chemokine-producing stromal network. In this model, immune cell loss emerges as an early and sensitive marker of irradiation, potentially initiating a cascade that progressively disrupts chemokine gradients and LN homeostasis. Despite lymphocyte depletion in the LN, we detected that regenerative mechanisms are already initiated at early time points following irradiation that contribute to partial regeneration of T cell subsets and a modest recovery in chemokine secretion.

However, this recovery is dose-limited. At 15-20 Gy, immune cell counts and chemokine levels remained suppressed, indicating a threshold beyond which LN function may be irreversibly compromised. These results suggest that irradiation first triggers lymphocyte depletion, which then leads to dysregulated CCL19/CCL21 signaling and impaired lymphocyte homing—a self-reinforcing cycle of LN dysfunction. Saddawi-Konefka et al. recently demonstrated the functional importance of intact lymphatic trafficking for immunoradiotherapy efficacy, showing that both physical disruption of lymphatic channels and pharmacological blockade of lymphocyte egress abrogate the therapeutic response to combined radiation and immune checkpoint blockade^23^. Notably, IL-2 and IL-7 levels – important for T cell survival^19,21^—remained stable or slightly increased in the irradiated LN, further indicating that disrupted homing signals and not only growth and survival factors are relevant for T cell reconstitution. An additional consideration when interpreting T cell recovery is whether the observed rebound reflects true local proliferation or redistribution from peripheral lymphoid reservoirs. While increased Ki67 expression suggests proliferative activity in situ, re-entry of circulating lymphocytes into the partially recovering LN microenvironment may also contribute, particularly at lower irradiation doses where chemokine gradients begin to re-establish. Given the partial and dose-dependent recovery of CCL19 and CCL21, it is plausible that both mechanisms act in parallel.

To directly assess the functional competence of irradiated stromal cells, we employed in vitro stimulation with LTα1β2—a key physiological signal provided by activated T cells stimulating CCL19 secretion^24,25^. While sham-irradiated FRCs robustly responded with increased CCL19 secretion, irradiated FRCs failed to do so, even in response to low-dose irradiation (5 Gy). These results indicate that irradiation also directly interferes on the level of FRCs, and underscores the central role of FRCs not only in the physical LN structure but also as active mediators in the immune cell homing process.

This impairment might be linked to defective activation of the non-canonical NF-κB pathway, particularly the RelB–p52 axis, which is critical for FRC differentiation and for the expression of CCL19 and CCL21^15,26–28^. Ionizing irradiation is known to disrupt multiple cellular pathways through persistent DNA damage and oxidative stress, which can impair receptor signaling or interfere with transcriptional and post-transcriptional processes, to be further investigated in irradiated FRCs. Emerging evidence from human tumors indicates that LTβR-driven differentiation of CCL19⁺/CCL21⁺ FRC-like fibroblasts supports the development of intratumoral tertiary lymphoid structures, a feature associated with improved responsiveness to immune checkpoint blockade^29^. Our results suggest that even moderate doses of irradiation can disrupt this stromal program within DLNs, leading to sustained functional impairment and diminished immune cell recruitment.

An additional explanation for the reduced responsiveness of irradiated stromal cells could involve radiation-induced senescence. High radiation doses can cause persistent DNA damage that triggers cellular senescence, characterized by altered gene expression and acquisition of a senescence-associated secretory phenotype (SASP)^30,31^. Whether senescence contributes to the impaired CCL19 response observed in our study, or whether this reflects a more specific signaling defect, remains to be determined.

Telarovic et al. previously demonstrated structural damage to the DLN architecture by day 9 following irradiation, including disorganization of the FRC network and loss of stromal cell subsets^13^. In our study, we demonstrated that stromal activation markers (ICAM-1, VCAM-1, and SCA-1) were elevated as early as 2 days post-irradiation, though overt architectural disruption only becomes evident later on. Furthermore, this disruption may persist long-term, with pronounced architectural alterations still evident 100 days post-irradiation. Immunofluorescence analysis revealed a disrupted FRC network, loss of B cell follicle structure, and radiation-induced fibrosis characterized by increased collagen I deposition and altered PDPN expression. These findings support the concept that early stromal activation transitions into chronic structural dysfunction, likely impairing immune cell trafficking and LN functionality well beyond the initial radiation insult. Further studies may point to strategies to preserve the functionality of high-dose irradiated LNs.

An important limitation of our study is the use of healthy LNs in non-tumor-bearing mice rather than bona fide tumor-draining lymph nodes. The immunological milieu of DLNs differs substantially from healthy LNs, containing tumor antigen-loaded dendritic cells, pre-activated T cell populations, altered chemokine gradients, and pre-existing architectural remodeling^11,32^. These baseline differences may modulate radiation responses, and the dose thresholds we define here may differ quantitatively in the tumor-draining context. However, we believe our reductionist approach was necessary to isolate direct radiation effects from the complex and variable tumor-induced alterations that would have introduced substantial heterogeneity.

Another limitation of this work is the exclusive use of female mice. This approach was chosen to decrease the anatomical variability, subsequently increasing the robustness of the radiotherapy treatment plans in our study. However, sex-specific differences can influence immune outcomes, and the generalizability of these results to male mice remains to be determined.

Overall, our data demonstrate that radiotherapy induces a dose-dependent breakdown of LN function, marked by lymphocyte loss, disrupted chemokine signaling, and stromal dysfunction. These findings support and extend earlier studies showing that irradiation timing critically influences radioimmunotherapy outcomes^13^. Given the DLNs’ central role in initiating adaptive immunity^8^, our results argue for carefully tailored radiation strategies – such as spatial or temporal separation of tumor and DLN irradiation—to avoid undermining immune responses. Future research, particularly in human models, should investigate molecular mechanisms of stromal damage and explore interventions to preserve DLN function and enhance synergy with immunotherapy.

## Methods

### Ethics approval

All animal experiments were conducted in compliance with Swiss federal and cantonal animal welfare regulations and received approval from the Cantonal Veterinary Office Zurich (license number ZH141/2023).

### Study design

The aim of this study was to investigate radiation-induced functional and structural changes in the lymph node, in order to understand how communication between tumors and DLNs is affected by radiotherapy. The study was conducted using female mice, which were either treated with radiotherapy or sham-irradiated. Female mice were exclusively used to reduce anatomical variability among subjects, thereby improving the consistency of radiotherapy outcomes. During the follow-up period, researchers handling the animals were blinded to the treatment groups. For unbiased data analysis, each mouse was randomly assigned a number by a person not involved in the analysis. Outliers were identified and removed using Grubbs’ test. Unless specified otherwise, data represent pooled or representative results from at least two independent experiments.

### Animals

Female C57BL/6J mice, aged 7–8 weeks, were purchased from Envigo (C57BL/6OlaHsd) and Janvier (C57BL/6JRj). The mice were housed under specific pathogen-free conditions at the University of Zurich’s animal facility. The facility maintains a 12-h light/dark cycle (lights

on: 07:00, lights off: 19:00), an ambient temperature of 21–24 °C and a humidity level of 35–70%. All interventions were performed under anesthesia using isoflurane (Attane, Piramal Ltd.) at an oxygen flow rate of 1 L/min, with 4.5% isoflurane used for induction and 1.5% for maintenance. At the designated experimental endpoints, mice were euthanized by CO2 inhalation at a flow rate of 2.5 L/min, and death was ensured by exsanguination via cardiac puncture.

### Small animal irradiation

Radiotherapy was performed using the X-RAD SmART small animal image-guided radiation platform (Precision X-Ray Inc.) in conjunction with the SmART-ATP treatment planning software (SmART Scientific Solutions B.V.). Treatment planning followed an established protocol^13,33^. Briefly, prior to each irradiation, the anesthetized mice underwent CT imaging to enable precise target identification and the creation of individualized treatment plans. Lymph nodes were irradiated via a posterior-anterior (PA) approach using a rectangular 8 × 12 mm collimator. Detailed information on beam characteristics, imaging parameters, and quality assurance measures is described in ref. ^34^.

### Flow cytometry

For immune cell analysis, axillary, brachial, and inguinal lymph nodes were harvested and kept on ice in a flow cytometry buffer (PBS containing 2% FBS and 2 mM EDTA) until further processing. Lymph nodes were disrupted into small fragments with two 26 G needles and digested in 500 μL of dissociation buffer (DMEM with 10% FBS, 0.5 mg/mL DNase I (Roche), and 1 mg/mL collagenase D (Roche)) per sample at 37 °C for 15 min. After digestion, tissues were filtered through a 70 μm cell strainer using a syringe plunger to obtain single-cell suspensions. Cell counts were determined using the EVE automatic cell counter (NanoEntek). Cells were then incubated with an extracellular staining mix at 4 °C for 30 min, followed by fixation and permeabilization with the Foxp3/Transcription Factor Staining Buffer Set (eBioscience) according to the manufacturer’s guidelines. Intracellular staining was performed overnight.

For stromal cell analysis, brachial, axillary, and inguinal lymph nodes were collected and kept on ice in RPMI 1640 medium (Gibco) supplemented with 2% FBS until further processing. Lymph nodes were disrupted into small fragments using two 26 G needles, then transferred into 1 mL of dissociation buffer per sample. The dissociation buffer consisted of RPMI 1640 with 2% FBS, 20 mM HEPES (pH 7.2; Lonza), 200 μg/mL collagenase P, 30 μg/mL dispase, and 10 μg/mL DNase I (all from Roche). Tissues were enzymatically digested for 45 minutes at 37 °C, with periodic resuspension and supernatant collection every 15 min. Stromal cells were enriched using magnetic anti-CD45 and anti-TER119 MicroBeads (Miltenyi Biotec). After a 20-minute incubation at 4 °C, samples were passed through MACS LS columns to isolate the CD45^-^ TER119^-^ stromal fraction. The resulting single-cell suspensions were then stained with the viability dye eFluor 780 (Invitrogen) for 20 min at 4 °C, followed by surface antibody staining for 30 minutes at 4 °C.

The antibodies utilized for flow cytometry are listed in Supplementary Table 1. Immune cell data were acquired using the Aurora spectral flow cytometer (Cytek) with SpectroFlo v3.0 software, while stromal cell data were acquired using the FACSymphony (BD Biosciences) with FACSDiva v8.0.1. Data were analyzed using FlowJo v10.8 software (BD Biosciences). Details on the quantitative analysis of the flow cytometry data can be found in the “Statistical analysis” section below. The gating strategy for LN immunophenotyping is shown in Supplementary Fig. 1.

### Multiplex cytokine analysis

Axillary, brachial, and inguinal lymph nodes were snap-frozen in liquid nitrogen and stored at −80 °C until further analysis. Proteins were extracted using the ProcartaPlex cell lysis buffer (Invitrogen) supplemented with the Halt protease and phosphatase inhibitor cocktail (Thermo Scientific) and processed with the BioMasher Standard disposable microhomogenizer (TaKaRa).

Lymph node lysates were prepared at dilution ratios of 1:1000 for CCL19, 1:10000 for CCL21, and 1:1 for IL-2 and IL-7 quantifications. Chemokine levels were determined using the appropriate Mouse ProcartaPlex Simplex kit (Invitrogen) in accordance with the manufacturer’s protocol. Data acquisition was performed using the Bio-Plex 200 system (Bio-Rad).

For analysis of cell culture supernatants, samples were subjected to measurement without prior dilution using the appropriate Mouse ProcartaPlex Simplex Kit (Invitrogen).

### Immunofluorescence microscopy

Axillary, brachial, and inguinal lymph nodes were harvested from mice at the indicated time points after irradiation (15 Gy delivered to each node) and fixed in freshly prepared 4% paraformaldehyde (Merck Millipore) at 4 °C for 24 h with gentle agitation. Following fixation, the tissues were rinsed with PBS containing 2% FBS and 0.1% Triton X-100 (Sigma) and embedded in 4% low-melting agarose (VWR International). The lymph nodes were then sectioned into 40 µm slices using a VT1200 vibratome (Leica). Tissue sections were blocked in PBS supplemented with 10% FBS, 1 mg/mL anti-Fcγ receptor (BD Biosciences), and 0.1% Triton X-100 (Sigma), then stained overnight at 4 °C with the appropriate antibodies in PBS containing 2% FBS and 0.1% Triton X-100 (Sigma). Unconjugated antibodies were stained with secondary antibodies.

All antibodies used for immunofluorescence are detailed in Supplementary Table 2. Confocal imaging was conducted with an LSM-980 confocal microscope (Carl Zeiss), and images were acquired and processed using ZEN 2010 software (Carl Zeiss). For image analysis, Imaris v9.2.1 (Oxford Instruments) was utilized. For quantification of COL1, ACTA2, and PDPN, tissue sections of lymph nodes were stained using antibodies against COL1, ACTA2, and PDPN, and T cell zones were imaged. The arithmetic mean intensity of COL1, ACTA2, and PDPN was used for quantification. For each assessed lymph node, a minimum of two images of T-cell zones per LN section was used for analysis.

### Lymph node stromal cell isolation and cultivation

Stromal cells were isolated using the same enzymatic digestion protocol applied for flow cytometry. The resulting single-cell suspension was centrifuged at 1200 rpm for 5 minutes, and the cell pellet was resuspended in 1 mL MACS buffer (PBS containing 2% FBS and 1 mM EDTA). Cells were washed with complete medium (RPMI 1640 (Gibco) supplemented with 10% FBS, 1% penicillin/streptomycin (Sigma), and 16 μg/mL gentamicin (Thermo Scientific)), then seeded into gelatin-coated 12-well plates, and cultured in complete medium at 5% CO_2_/37 °C. After 48 hours, non-adherent cells and debris were removed by replacing the medium. Once adherent cells reached confluency, the culture medium was removed, and the monolayer was washed with PBS. Cells were then detached using 0.05% trypsin-EDTA (Cell Biologics) and reseeded at a 1:2 ratio into new plates in fresh medium. Stromal cells used in experiments were at a maximum of passage 3.

### In vitro stimulation of stromal cells

Cultured stromal cells were treated with 100 ng/mL lymphotoxin-α1β2 (R&D Systems) in serum-reduced medium (RPMI 1640 supplemented with 1% FBS) or with medium alone as controls. After 1 h of stimulation, cells were irradiated at 6.63 Gy/min (RS-2000 225 kV irradiator, Rad Source), or sham-irradiated, and then incubated for an additional 24 or 48 h. Culture supernatants were collected and stored at –80 °C for subsequent multiplex cytokine analysis. Cells were lysed in 400 µL TRIzol reagent (Invitrogen) per well, and lysates were stored at –20 °C until further RNA extraction.

### Real-time quantitative PCR

RNA was extracted using the PureLink RNA Mini Kit (Thermo Scientific). Complementary DNA (cDNA) was synthesized with the High-Capacity cDNA Reverse Transcription Kit (Applied Biosystems), and quantitative PCR was performed using the KAPA SYBR® FAST qPCR Kit (Kapa Biosystems) on a LightCycler® 480 system (Roche). Gene expression levels were determined using Ccl19 (QT02532173, Qiagen), Tgfβ1 (QT00145250, Qiagen) and Hprt (QT00166768, Qiagen) primers.

### Statistical analysis

All statistical analyses were performed using Prism v10.1 (GraphPad). Data were tested for normality with the Shapiro–Wilk test. For datasets with a normal distribution, comparisons between treatment groups were made using the two-sided unpaired *t*-test (for two groups) or one-way ANOVA with Holm–Sidak’s multiple comparisons test. Non-normally distributed data were analyzed using the two-sided Mann–Whitney test. Statistical results were derived exclusively from three or more biological replicates, with the alpha level set at 0.05. All p-values are reported as precise numbers.

## Supplementary information

SupplementaryFiles.

## Data Availability

All data generated or analysed during this study are included in this published article and its supplementary information files.
