# Supplementary material for: Dynamic alterations in the irradiated draining lymph node
Source: NPJ Precis Oncol. 2026 Jul 11;10:423. doi: 10.1038/s41698-026-01610-y (PMC13736964; doi:10.1038/s41698-026-01610-y)
Supplement: Supplementary file 1 [file 41698_2026_1610_MOESM1_ESM.pdf]

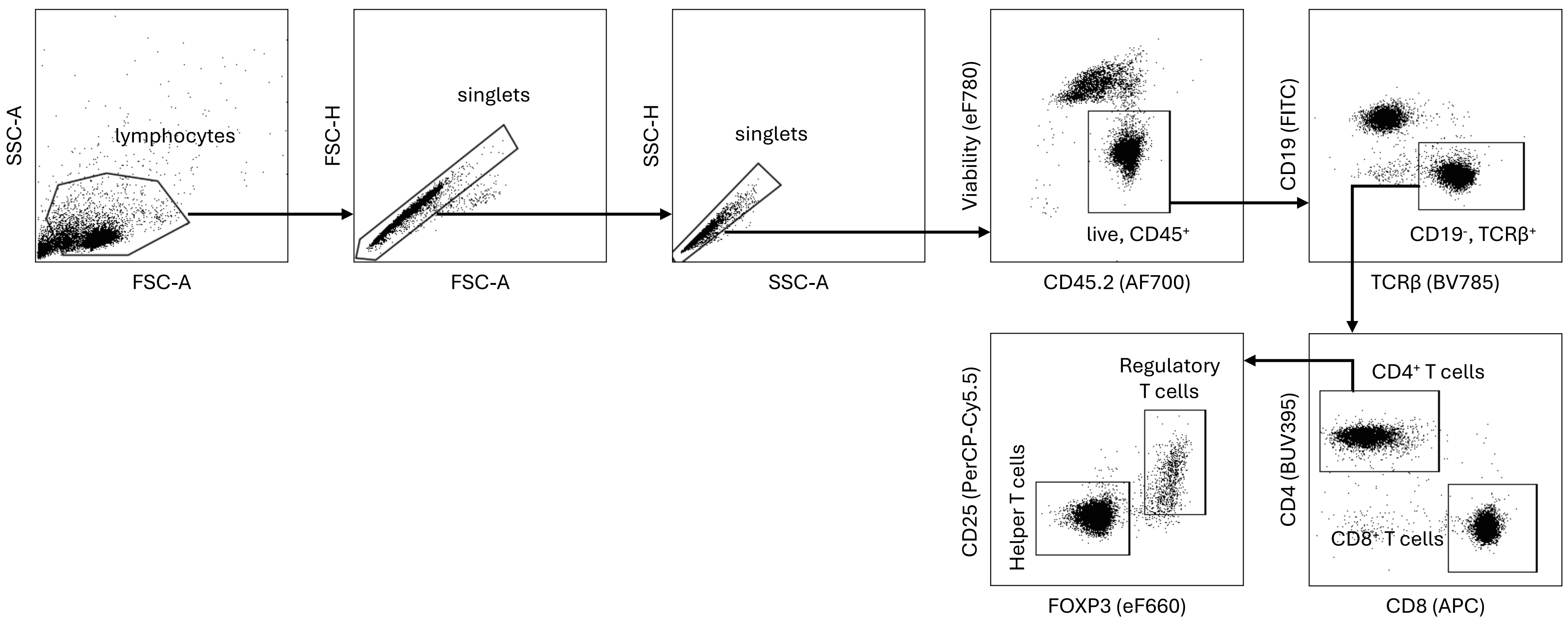

Supplementary Figure 1: Representative gating strategy for lymph node immunophenotyping. Corresponding data is shown in Figure 1A-B, Figure 2A-B, and Figure 6A-B.

Day 2 after IR

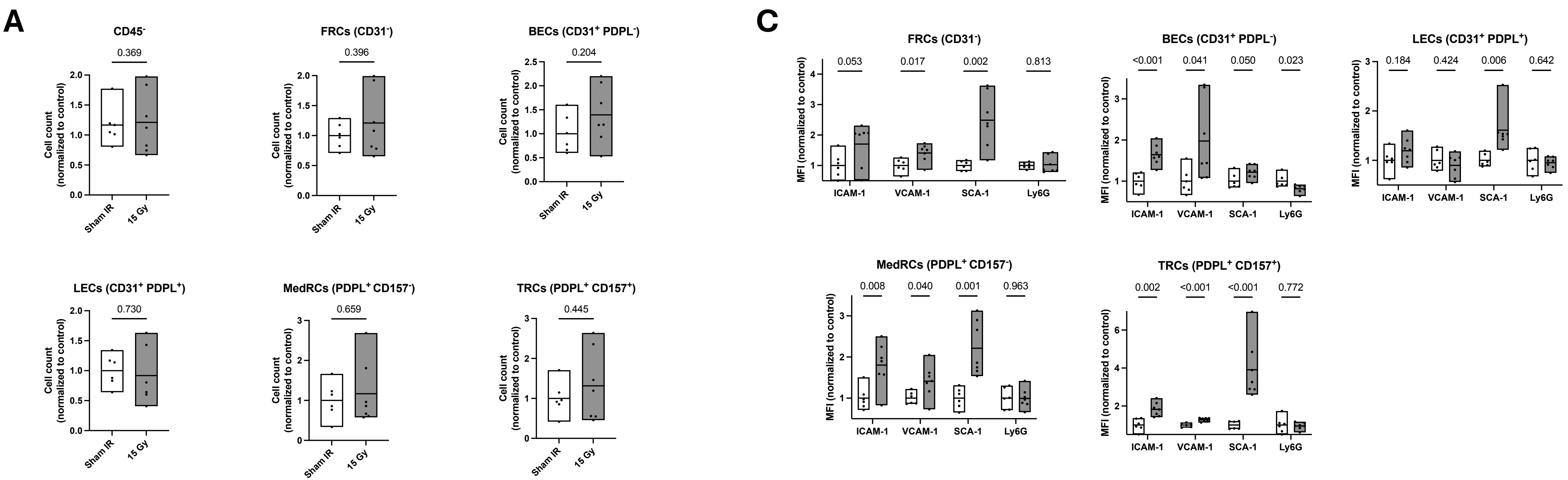

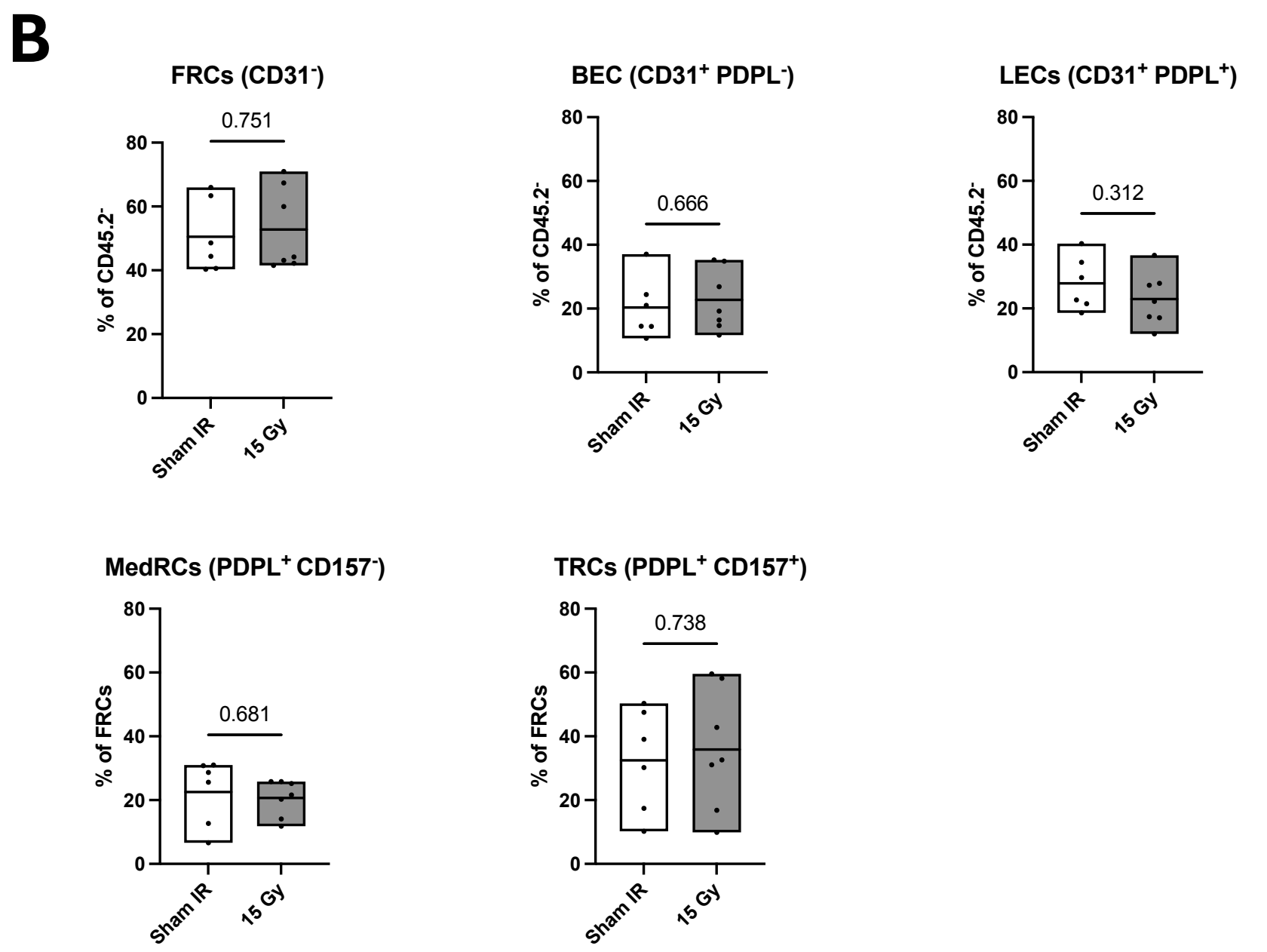

Supplementary Figure 2: Early stromal activation in irradiated lymph nodes. Brachial, axillary, and inguinal LNs on both sides of C57BL/6 mice were irradiated with 15 Gy and harvested after 2 days. **A-B**) Stromal cell composition of the LNs. $CD45^-$ cells, fibroblastic reticular cells (FRCs), lymphatic endothelial cells (LECs), blood endothelial cells (BECs), and FRC subtypes, including medullary reticular cells (MedRCs) and T zone reticular cells (TRCs), are shown as cell counts (**A**) and as percentages of $CD45^+$ or $CD31^-$ cells (**B**). Gating strategy as described by Telarovic et al.[13] **C**) Expression of activation markers ICAM-1, VCAM-1, SCA-1, and Ly6G on major stromal cells subgroups, expressed as the geometric mean of the fluorescence intensity (MFI), normalized to the average MFI value of sham-irradiated mice (grey). n = 6 mice per group. Each dot represents one individual mouse. Floating bars span from the minimum to the maximum value within each group. Line indicates the mean value. Statistics: two-sided unpaired t-test.

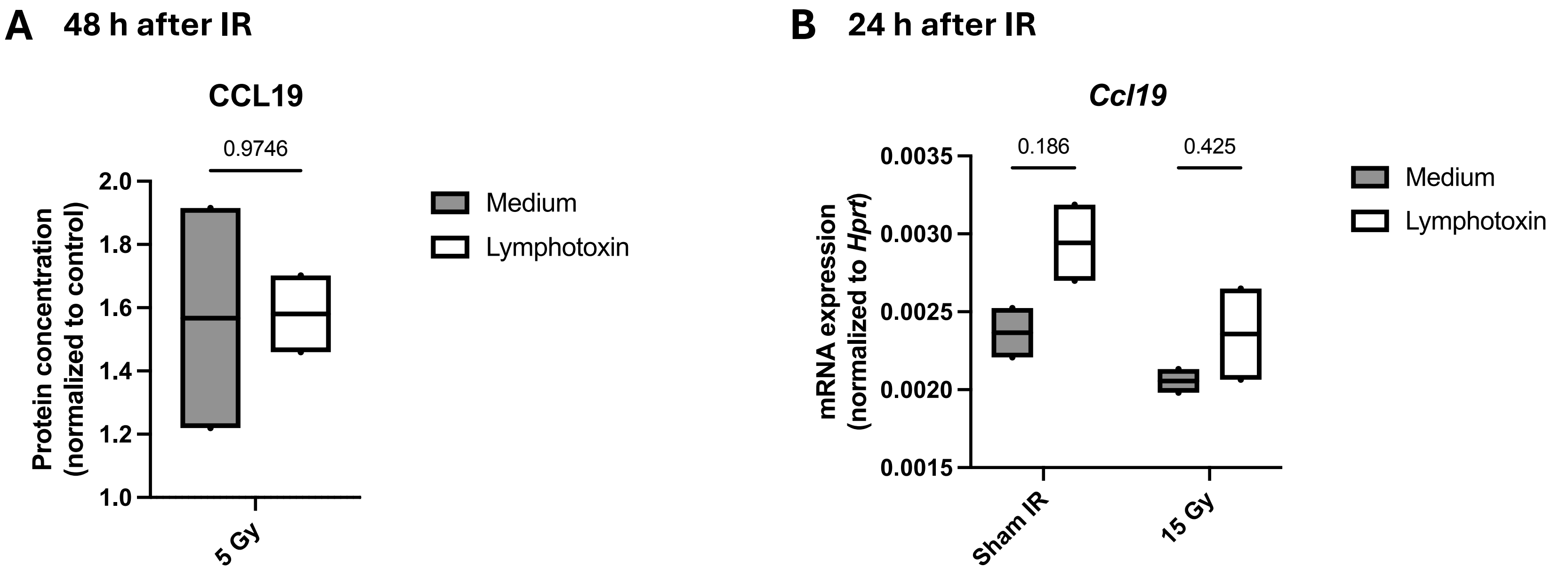

Supplementary Figure 3: LTα1β2-induced CCL19 protein secretion and *Ccl19* expression in irradiated lymph node stromal cells.

Isolated LN stromal cells were treated with lymphotoxin-α1β2 (white) or serum-reduced medium alone (grey) and irradiated with 5 (**A**) or 15 Gy (**B**). **A**) CCL19 protein concentration in the supernatant 48 h post-irradiation, expressed relative to the average value of sham-irradiated, serum-treated cells. **B**) *Ccl19* mRNA expression in the cells, normalized to *Hprt*, after 24 h. Each dot represents one individual experiment; n = 2. Floating bars span from the minimum to the maximum value within each group. Line indicates the mean value. Statistics: two-sided unpaired t-test.

Target	Clone	Fluorochrome	Dilution	Reference	Distributor
CD106 (VCAM-1)	429	BV711	400	740675	BD Biosciences
CD157	BP-3	BV786	200	741012	BD Biosciences
CD19	6D5	FITC	200	115506	Biolegend
CD21/35	7E9	APC	200	123412	Biolegend
CD31	MEC13.3	BUV805	200	741939	BD Biosciences
CD4	GK1.5	BUV395	200	565974	BD Biosciences
CD45	30-F11	BUV496	200	749889	BD Biosciences
CD45.2	104	AF700	200	109822	Biolegend
CD54 (ICAM-1)	3E2	BV421	200	564704	BD Biosciences
CD8β	YTS156.7.7	APC	200	126614	Biolegend
FOXP3	FJK-16s	eF660	200	50-5773-82	eBioscience
Ki67	16A8	BV605	200	652413	Biolegend
Ly6A/E (Sca1)	D7	BV510	400	108129	Biolegend
Ly6C	AL-21	PerCP-Cy5.5	200	560525	BD Biosciences
MAdCAM-1	MECA-367	BUV615	200	751510	BD Biosciences
PDPN	36899	PE-Cy7	800	127412	Biolegend
TCRβ	H57-597	BV785	200	109249	Biolegend
CD16/32 (Fc block)	93	-	100	101302	Biolegend
Fixable Viability dye eF780			1000	65-0865-18	eBioscience

Supplementary Table 1: Antibodies used for flow cytometry

Target	Clone	Fluorochrome	Dilution	Reference	Distributor
aSMA	1A4	Cy3	1000	C6198	Sigma-Aldrich
aSMA	1A4	eF660	1000	50-9760-82	eBioscience
B220	RA3-6B2	eF450	200	48-0452-82	Thermo Scientific
CD31	MEC13.3	AF594	400	102520	Biolegend
COL1	polyclonal	-	500	AB758	Sigma-Aldrich
LYVE1	ALY-7	eF660	200	50-0443-82	eBioscience
PDPN	8.1.1	-	300	127402	Biolegend
Anti-goat		AF488	1000	705-545-003	Jackson Immunoresearch
Anti-Syrian hamster		AF594	1000	307-585-003	Jackson Immunoresearch

Supplementary Table 2: Antibodies used for immunofluorescence

		Sham IR	2 Gy	5 Gy	10 Gy	15 Gy	20 Gy
Cell count of CD8+ T cells	Exp. 1	1909440		2267785,2	827316	1324224	1129928,8
		1817515,2		1149395,5	1509148,3	1385100	933701
		1702641,2		818748	658896	650423,4	1406033,5
		1960602,8		1049610	918793,6	978541,2	1073856
	Exp. 2	1631108,4		616032	640016	518918,4	804650
		1504142,5		878450,4	1003371	614628	252230,4
		970362		788036,4	883116	728870,4	247841,88
		1768676		1204018	689920	927744,4	725978,4
	Exp. 3	2334990,2	2034516,3			827996,4	
		1491891,2	1497434,4			1576908,2	
		2441220	2679091,2			1562362,5	
		1492464	1645395,6			802620	
	Exp. 4	2123812,5	1765400			1145794	
		3178560	2403984			711009	
		2532474	2046049,2			713090	
		1814467,2				1303502,4	
Cell count of Helper T cells	Exp. 1	1879488		1968266,4	873278	1076416	984911,2
		1659470,4		1055904	1280010,6	972648	657359
		1576296,8		676544,4	576534	504339	1097392
		1639300		849472,5	845485,6	698279,4	919632
	Exp. 2	1661426,4		598920	530400	454053,6	710600
		1579640		909043,2	951472,5	507312	231936
		970362		824903,6	803862	600998,4	217168,38
		1605413,6		1110749	658560	848365,2	630909,8
	Exp. 3	2042039,4	2303226			618618	
		1416131,1	1121630,4			1210995,8	
		2271396	2567462,4			1186712,5	
		1290096	1428079,2			556920	
	Exp. 4	1815375	1892800			1001442	
		3448170	2528328			570414	
		2621556	2046049,2			538020	
		1709786,4				1303502,4	
Cell count of Regulatory T cells	Exp. 1	247852,8		283473,15	93893,8	144038,4	153476,96
		204799,72		136937,55	168297,69	134816,4	103000,2
		181093,64		87045,84	63830,55	67477,08	159807,71
		180323		104516,25	113383,04	93578,94	139372,8
	Exp. 2	191003,4		80084,16	79913,6	62800,92	115472,5
		178871		128926,8	155695,5	75771,6	34790,4
		130895,64		124426,8	121145,4	86526,72	33372,768
		185710,98		156013,6	95424	124526,12	95932,86
	Exp. 3	286057,84	351881,75			107544,36	
		193479,64	153212,4			209964,02	
		338586,6	389460,48			191240	
		218178	229734,48			91318,5	
	Exp. 4	235293,75	244790			156080,6	
		388806	364742,4			66682,2	
		351237,6	278586,36			73444	
		249489,24				181097,28	

Supplementary Table 3:
Absolute numbers of CD8+ T cells, helper T cells (CD4+ FOXP3-) and regulatory T cells (CD4+ FOXP3+) in irradiated LNs. Data correspond to Figure 1A (day 2 post-irradiation).

		Sham IR	2 Gy	5 Gy	10 Gy	15 Gy	20 Gy
Cell count of CD8+ T cells	Exp. 1	1687838,4<br>1694926,8<br>1084723,2		1132396,2<br>1285038,3<br>583718,4<br>1300515	1052543,2<br>1371600<br>1135456<br>711450	1014420<br>1159344<br>324597	131016<br>428430,6<br>216007,26<br>409059
	Exp. 2	1792425,6<br>498482,1<br>1526170,8<br>779032,8		961925<br>1266220,8<br>1256154,6<br>1826916	863951,4<br>764250,3<br>883048<br>406177,2	916852,5<br>1462734<br>1128691,2<br>956826	513658,8<br>114094,4<br>499232,4<br>103024,95
	Exp. 3	3633476<br>1266408<br>3155064<br>2557846,4	2081816<br>1996215,9<br>1155465<br>1678158,4			1438866<br>609367,5<br>1326351,6<br>719239,5	
	Exp. 4	2481022,5<br>1343151,8<br>2352105<br>2461316,1	1855854,8<br>1575201<br>1647507,4<br>1916291			790173,9<br>1158769,8<br>768240<br>757816,8	
Cell count of Helper T cells	Exp. 1	1762702,2<br>1782363,5<br>1035417,6		1112389,2<br>1252088,6<br>545209,2<br>1431975	946503,4<br>1301400<br>851592<br>682465	954477<br>1212480,6<br>307443,5	109592<br>368760,6<br>189174,06<br>361651,5
	Exp. 2	1799956,8<br>464829,3<br>1394034,8<br>661861,2		908975<br>1166630,4<br>1085998,2<br>1651104	671962,2<br>684307,8<br>735188,8<br>352367,4	725679<br>1148374,5<br>995904<br>842742,9	477122,4<br>90053,08<br>397762,4<br>92512,2
	Exp. 3	3416336<br>1170468<br>3348796<br>2469644,8	1911485,6<br>1804439,7<br>1093840,2<br>1515230,4			1322035<br>559282,5<br>1180998<br>635607	
	Exp. 4	2510211<br>1395033,2<br>2432902,5<br>3323296	2057977,6<br>1239614,7<br>1533624,4<br>1803568			606160,8<br>1051096,5<br>570240<br>697725,6	
Cell count of Regulatory T cells	Exp. 1	233438,94<br>243477,58<br>154080		122442,84<br>149685,78<br>72964,8<br>137094	144135,58<br>170640<br>113951,12<br>89853,5	134180,1<br>150231,66<br>43147,65	20558,8<br>49526,1<br>24082,797<br>47542,95
	Exp. 2	267357,6<br>66043,62<br>188954,48<br>103237,68		131933,75<br>153179,52<br>143632,02<br>214796,4	117777,99<br>90814,68<br>121162,4<br>66828,3	120556,35<br>214919,25<br>166932,48<br>103778,82	81884,52<br>15973,216<br>63114,34<br>17577,318
	Exp. 3	427042<br>146788,2<br>420675,2<br>314218,2	321735,2<br>267614,97<br>138142,26<br>213435,68			201687,2<br>76240,5<br>201072,48<br>90657,63	
	Exp. 4	311344<br>195419,94<br>249574,5<br>305327,82	277459,48<br>189024,12<br>207267,06<br>188160,7			120149,73<br>157408,11<br>100980<br>105493,44	

Supplementary Table 4:
Absolute numbers of CD8+ T cells, helper T cells (CD4+ FOXP3-) and regulatory T cells (CD4+ FOXP3+) in irradiated LNs. Data correspond to Figure 2A (day 9 post-irradiation).

		Sham IR	15 Gy
Cell count of CD8+ T cells	Exp. 1	2299050	594184,8
		3305549,6	529385
		1476035,2	454769,2
	Exp. 2	3401073	514324,8
		2189721,6	627198
		2758509,6	3568992
Cell count of Helper T cells	Exp. 1	1762605	402996
		2683472	465150
		1188425,4	409102
	Exp. 2	2529003	409122
		1561190,4	495924
		2108778	2587519,2
Cell count of Regulatory T cells	Exp. 1	284315,85	79849,44
		536694,4	104105
		199698,88	75541,16
	Exp. 2	481091,95	102280,5
		396380,16	138858,72
		418335,96	701901,76

Supplementary Table 5:
Absolute numbers of CD8+ T cells, helper T cells (CD4+ FOXP3-) and regulatory T cells (CD4+ FOXP3+) in irradiated LNs. Data correspond to Figure 6A (day 100 post-irradiation).
